# Supplementary material for: Utilizing spent mushroom substrate for rearing black soldier fly (Hermetia illucens) larvae: enhancing fertilizer efficiency and improving animal feed quality for sustainable agriculture
Source: PeerJ. 2025 Jun 19;13:e19590. doi: 10.7717/peerj.19590 (PMC12182726; doi:10.7717/peerj.19590)
Supplement: Supplemental Information 2 — AC, Agrocybe cylindracea; LP, Lentinus polychrous; PP, Pleurotus pulmonarius; NF, non-fermented and F, fermented. [file peerj-13-19590-s002.docx]

**Table S1.** Composition, harvest cycles, and utilized duration of the tested mushroom media.

AC = *Agrocybe cylindracea*, LP = *Lentinus polychrous*, PP *= Pleurotus pulmonarius*, NF = non-fermented and F = fermented.

| SMS type | Composition | Utilized duration |
| --- | --- | --- |
| LP | Rubberwood sawdust, rice bran, gypsum, magnesium sulfate, calcium oxide | 5 months |
| PP | Rubberwood sawdust, rice bran, gypsum, magnesium sulfate, tapioca starch, calcium oxide, carbamide, volcanic soil | 12 months |
| AC | Rubberwood sawdust, gypsum, rice bran, corn meal | > 12 months |
