# Supplementary material for: Utilizing spent mushroom substrate for rearing black soldier fly (Hermetia illucens) larvae: enhancing fertilizer efficiency and improving animal feed quality for sustainable agriculture
Source: PeerJ. 2025 Jun 19;13:e19590. doi: 10.7717/peerj.19590 (PMC12182726; doi:10.7717/peerj.19590)

**Table S2.** Substrate moisture (%) of various tested substrates measured every three days until day 24. The green and red colors represent the highest and lowest values, respectively, with the gradient between the two colors displaying the moisture trend.

VEG = control, AC = *Agrocybe cylindracea*, LP = *Lentinus polychrous*, PP *= Pleurotus pulmonarius*, NF = non-fermented and F = fermented


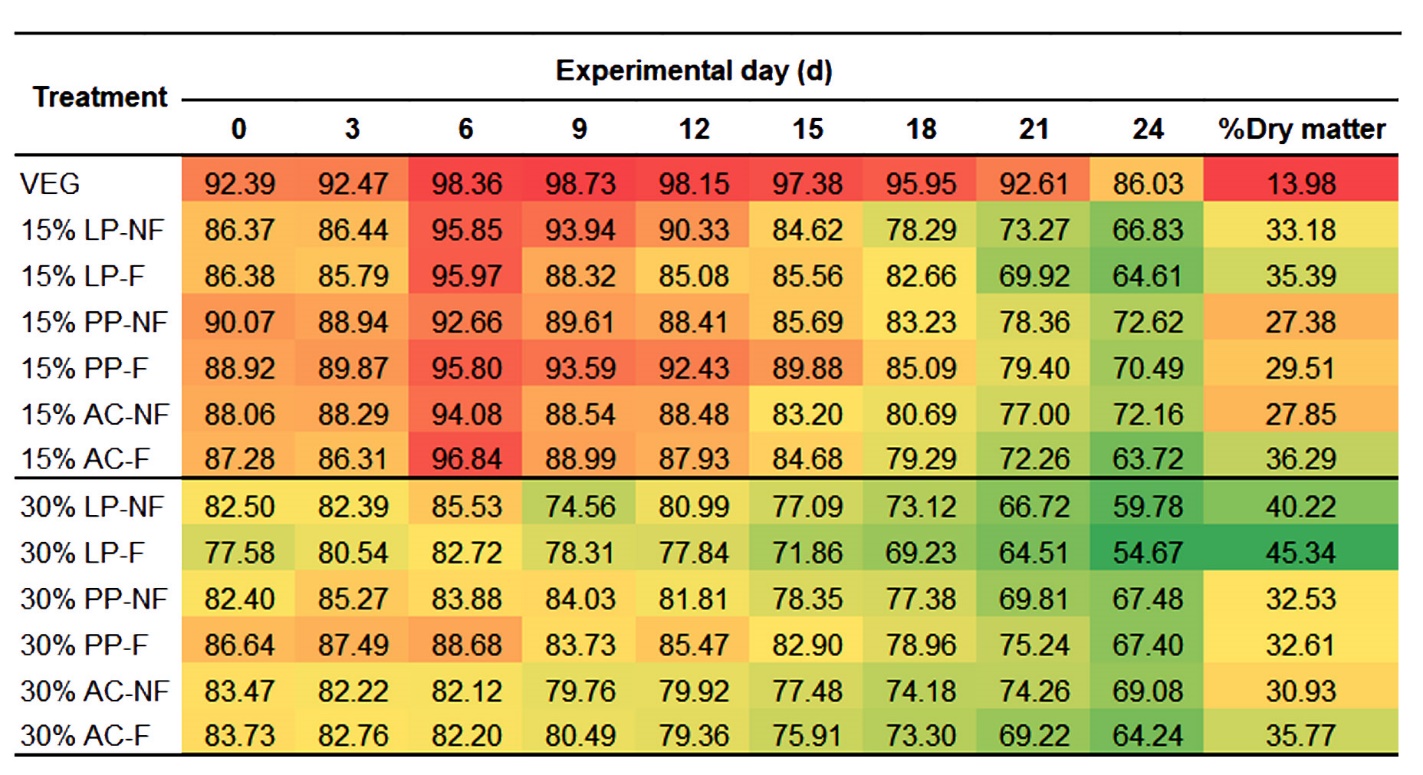

Supplement: Supplemental Information 3 — The green and red colors represent the highest and lowest values, respectively, with the gradient between the two colors displaying the moisture trend. VEG, control; AC, Agrocybe cylindracea; LP, Lentinus polychrous; PP, Pleurotus pulmonarius, NF, non-fermented and F, fermented. [file peerj-13-19590-s003.docx]
